# Effect of Substituent Groups on the Adsorption Efficiency of Phenols by Activated Carbon Developed by Hydrothermally Treated Phyllanthus Emblica Fruit Stone

**DOI:** 10.3390/toxics12120874

**Published:** 2024-11-30

**Authors:** Suhas, Sarita Kushwaha, Monika Chaudhary, Shubham Chaudhary, Vaishali Tyagi, Isabel Pestana da Paixão Cansado, Mohammad Hadi Dehghani

**Affiliations:** 1 Department of Chemistry, Gurukula Kangri (Deemed to Be University), Haridwar 249404, India; saritakushwaha31@gmail.com (S.K.); shubhamchaudhary89@yahoo.com (S.C.); tyagivaish1@gmail.com (V.T.); 2 Department of Chemistry, Hariom Saraswati P.G. College, Dhanauri, Haridwar 247667, India; monikachoudry@gmail.com; 3 MED—Mediterranean Institute for Agriculture, Environment and Development & Change—Global Change and Sustainability Institute, Department of Chemistry and Biochemistry, School of Science and Technology, University of Évora, Rua Romão Ramalho, nº 59, 7000-671 Évora, Portugal; 4 Department of Environmental Health Engineering, School of Public Health, Tehran University of Medical Sciences, Tehran 1416634793, Iran; hdehghani1388@gmail.com; 5 Center for Solid Waste Research, Institute for Environmental Research, Tehran University of Medical Sciences, Tehran 1416634793, Iran

**Keywords:** activated carbon, *Phyllanthus emblica* fruit stone, adsorption, 4-nitrophenol, 4-chlorophenol, phenol

## Abstract

In this study, the novel activated carbon developed from *Phyllanthus emblica* fruit stone, through hydrothermal treatment at low pressure and temperature, was utilized for the removal of 4-nitrophenol, 4-chlorophenol, and phenol from water. The activated carbon produced (AC-HTPEFS) showed a well-developed porosity with a surface area of 569 m^2^ g^−1^ and a total pore volume of 0.342 cm^3^ g^−1^. The adsorption process was explored and efficiently applied regarding the removal of phenols and substituted phenols from wastewater. Thermodynamic analyses indicated that the adsorption process was endothermic and spontaneous. To analyze the equilibrium data, different isotherm models were employed. The Langmuir model applied best, with maximum adsorption capacities of 0.463, 0.434, and 0.387 mmol g^−1^ at 25 °C for 4-nitrophenol (4-NP), 4-chlorophenol (4-CP), and phenol, respectively, regarding the AC-HTPEFS. The adsorption was mainly an endothermic process, and the results achieved were slightly higher than those obtained with a commercial activated carbon commonly used for this purpose.

## 1. Introduction

The adsorption of phenols on activated carbons (ACs) has been the subject of extensive research over the years. Phenols contain one or more hydroxyl groups (–OH) directly bonded to the aromatic ring [1]. The different substituents, such as -NO_2_, -Cl, -CH_3_, -NH_2_, etc., present on the phenolic ring significantly influence their removal from aqueous solutions. The number, chemical nature of these substituents, and their position on the ring significantly change the properties of the phenols [1]. Furthermore, different substituents attached to the phenolic ring influence the density of electrons and their distribution by withdrawing or donating electrons [2], which can affect the interaction between the adsorbate and adsorbent.

A diverse range of phenols and substituted phenols are frequently found in the wastewater from industries, viz. pharmaceutical, chemical, wood, petroleum, paper, dye, pesticide, rubber, etc. [3,4]. Most phenolic compounds are recognized as toxic and carcinogenic [5]. The toxicity of these compounds depends on the number and nature of the side groups present on the benzene ring [6]. Phenols are listed as priority toxic pollutants, and it becomes a major challenge to remove them from wastewater before being discharged. Both the US-EPA and European Union have designated some phenols, especially nitrophenols and chlorophenols, as priority pollutants. EU Directive 2455/2001/EC limits the allowable concentration of these substances in drinking water to 0.5 µg·L^−1^, with the individual concentration restricted to no more than 0.1 µg·L^−1^ [7]. Thus, owing to their limited biodegradability, high toxicity, and extensive distribution, phenols are necessarily removed from wastewater prior to their disposal into aquatic environments.

However, the removal of phenols and their derivatives from ground and surface water is very difficult owing to their high solubility and stability in water. Moreover, phenols and substituted phenols are resistant to the conventional water treatment methods [8]. Different technologies for their removal from wastewater have been discussed in the literature, including membrane filtration, biological degradation and ion exchange [9], reverse osmosis [10], electrochemical oxidation [11], photocatalytic degradation [12], and adsorption [13]. Among all these techniques, adsorption, especially using AC, is notably effective in removing phenols from aqueous solutions. Owing to their unique characteristics and versatility, ACs are well recognized for removing an array of gaseous- and aqueous-phase contaminants.

Various lignocellulosic materials have been used for the preparation of activated carbon (AC); however, this study focuses on the utilization of PEFS, a waste material also possessing medicinal values. The production of ACs from PEFS not only contributes to waste management but may also comprise some medicinal benefits. This study employs green technology, specifically hydrothermal carbonization (HTC), followed by the physical activation method.

In the present study, 4-NP, 4-CP, and phenol were selected for investigation to analyze the effect of the substituent groups on the aromatic ring on adsorption. To our knowledge, no investigations have been performed regarding the adsorption of these phenols onto AC-HTPEFS. This study focuses on the application of AC-HTPEFS for the removal of 4-NP, 4-CP, and phenol from aqueous effluents. Further, the effectiveness of AC-HTPEFS was evaluated by comparing it with a commercial activated carbon (CAC), a raw PEFS, and HTPEFS at 25 °C. The equilibrium data were analyzed by applying different isotherm models in order to obtain more insight regarding the adsorption mechanism and adsorption capacity. Furthermore, the adsorption rates were estimated by fitting the experimental data to different kinetics models, such as pseudo-first-order (PFO), pseudo-second-order (PSO), and intraparticle diffusion (IPD) models. This work will emphasize the role of the functional group in the removal of 4-NP, 4-CP, and phenol from aqueous solutions.

## 2. Materials and Methods

### 2.1. Materials

4-NP, 4-CP, and phenol were purchased from Sisco Research Laboratories (SRL) Pvt. Ltd. (Mumbai, India), Sigma Aldrich (St. Louis, MO, USA) and Merck (Darmstadt, Germany), respectively. All the chemicals used in this study were of AR grade and were utilized as received. Double distilled water was used for the preparation of all aqueous solutions. Standard CAC (surface area 635 m^2^ g^−1^) was procured from Fisher Scientific Mumbai (Mumbai, India).

### 2.2. Preparation of Adsorbents

The raw *Phyllanthus emblica* fruit stone (PEFS) was obtained locally. Raw PEFS was washed with distilled water, dried at 100 °C for 24 h, and crushed to obtain uniform particles. Further hydrothermal treatment of PEFS granules was carried out in an economical autoclave at a pressure of 15 psi and a temperature of 121 °C. The sample obtained, named HTPEFS, was further filtered, dried, and kept in a sealed container for further use. In addition, the AC was obtained by heating the HTPEFS in a muffle furnace in the presence of air (oxidizing atmosphere) at the temperature of 400 °C for 90 min. Further, the material obtained, named AC-HTPEFS, was kept in desiccators for cooling and stored in an airtight container.

### 2.3. Adsorbent Characterization

Elemental analyses were conducted to assess weight percent of C, H, N, and S in raw PEFS, HTPEFS, and AC-HTPEFS, performed using a Vario Micro CHNS Elemental Analyzer. The analyses of inorganic elements in the samples were conducted using an ICP, model Optima 7000 DV from Perkin Elmer (Waltham, MA, USA). The surface chemistry of the adsorbents used (raw PEFS, HTPEFS, and AC-HTPEFS) was investigated by Fourier-Transform Infrared Spectroscopy (FTIR) with a Perkin Elmer Paragon 1000 PC spectrophotometer, employing the KBr pellet method, as presented elsewhere [14].

Thermal gravimetric analysis (TGA) of raw PEFS and HTPEFS was carried out with Exstar TG/DTA 6300 analyzer using a procedure describe elsewhere [14]. X-ray diffraction (XRD) analysis of the samples (raw PEFS, HTPEFS, and AC-HTPEFS) was performed using a Rigaku Smart Lab diffractometer. Using a Quantachrome Instruments NOVA model, nitrogen adsorption–desorption isotherms were recorded to determine the surface area and total pore volume of the activated carbon. The Brunauer–Emmett–Teller (BET) method was used to calculate the specific surface areas. Field Emission Scanning Electron Microscopy (FE-SEM) was used to analyze the surface morphology of raw PEFS, HTPEFS, and AC-HTPEFS by FE-SEM, Tescan Mira 3, and procedure described elsewhere [14].

### 2.4. Adsorption Studies

Adsorption studies were performed using a batch method. For these experiments, 0.01 g of each adsorbent (PEFS, HTPEFS, AC-HTPEFS, and CAC) were added into a stoppered glass tube containing 10.0 mL of solutions with varying concentrations of adsorbates (4-NP, 4-CP, and phenol). The mixtures were agitated in a thermostatic shaker at room temperature until the equilibrium was reached. The concentration of unadsorbed phenols (4-NP, 4-CP, and phenol) was evaluated using a spectrophotometer (Agilent Cary 60 UV–Vis spectrophotometer) at the maximum absorbance wavelengths for each adsorbate (4-NP—317 nm; 4-CP—280 nm; phenol—270 nm).

The amount of 4-NP, 4-CP, and phenol adsorbed was quantified by employing the following equation:

(1) $$q_{e}=\frac{\left(C_{0}-C_{e}\right)V}{W}$$

where W represents mass (g) of the adsorbent (PEFS, HTPEFS, AC-HTPEFS, and CAC) used, V represents volume of solution used (L), q_e_ represents amount of phenols adsorbed (mol g^−1^), and C_0_ and C_e_ represent the initial and equilibrium concentrations of phenols (mol L^−1^) in the solution, respectively. Furthermore, the pH of all solutions in contact with adsorbents was found to be in the range 5–6.0.

## 3. Results and Discussion

### 3.1. Adsorbent Characterization

The HTPEFS was utilized for the preparation of AC-HTPEFS in an oxidizing atmosphere (air) at 400 °C, which exhibited the maximum surface area (569 m^2^ g^−1^). In our previous publication [14], we provided a comprehensive characterization of the material. For the sake of clarity and context, we have summarized some key aspects here. The elemental analysis reveals that the carbon content in the raw PEFS, HTPEFS, and AC-HTPEFS increases from 46.46% to 47.91% and then to 70.23%, respectively. This increase is owing to the removal of noncarbon elements as volatile gaseous compounds. The hydrothermal treatment of PEFS reduces its inorganic content, which slows the pyrolysis rate and enhance the activation process of HTPEFS in a highly oxidizing environment. The thermal gravimetric analysis reveals that a slight reduction in the mass of raw PEFS and HTPEFS occurred near 100 °C, which was associated with the loss of free water. The initial decomposition temperatures for raw PEFS and HTPEFS occur between 226–355 °C and 237–328 °C, respectively, which are associated with the partial degradation of cellulose and breakdown of hemicellulose. The subsequent decomposition stages for raw PEFS and HTPEFS occur in the ranges of 355–490 °C and 328–469 °C, respectively, due to the degradation of lignin and cellulose. The TGA results for HTPEFS suggest that the optimal temperature range of 330–470 °C is ideal for producing AC-HTPEFS. The XRD patterns of PEFS and HTPEFS present peaks at 2θ = 16° and 22°, which confirm the presence of the crystalline structure of cellulose. Conversely, the XRD pattern of AC-HTPEFS reveals a broader peak at 2θ = 22° to 26°, reflecting its amorphous nature, along with less pronounced peaks at 43°, indicating minimal graphitization. The FTIR studies [14] indicate that the spectra of PEFS and HTPEFS are quite similar, with no additional functional groups appearing after the hydrothermal treatment. Only a small decrease in the peak intensity, mainly in the C=O stretching bands, was observed. Notably, the C=O stretching bands associated with the carboxyl, aldehyde, ketone, and ester groups, as well as the bands related to alcohols, are less pronounced in HTPEFS. The FTIR spectrum of AC-HTPEFS reveals a marked reduction in the number of peaks compared to raw PEFS and HTPEFS, with the bands at 1734 cm^−1^, 1255 cm^−1^, and 1078 cm^−1^ nearly vanishing. However, peaks at 1629 cm^−1^ and 1413 cm^−1^ remain present in AC-HTPEFS, HTPEFS, and raw PEFS. In the case of the FE-SEM analysis, the raw PEFS showed a relatively compact surface, whereas HTPEFS exhibited slight surface destruction. However, in AC-HTPEFS, the surface shows the presence of cracks and cervices owing to the release of volatile organic compounds. The nitrogen adsorption enabled obtaining a relatively low surface area for the raw PEFS and HTPEFS. However, the AC-HTPEFS prepared at 400 °C was characterized by a Type I isotherm. The AC-HTPEFS exhibited a significantly high surface area (569 m^2^ g^−1^) and a total pore volume and pore width of 0.342 cm^2^ g^−1^ and 3.61 nm, respectively. The mean pore size of the AC-HTPEFS suggests that the hydrothermal treatment is an ecofriendly and sustainable pretreatment for producing activated carbons that are effective regarding the removal of phenols from wastewater.

### 3.2. Effect for Different Parameters on Phenolic Compounds Adsorption

#### Effect of Contact Time and Initial Concentration

**Figure 1:**
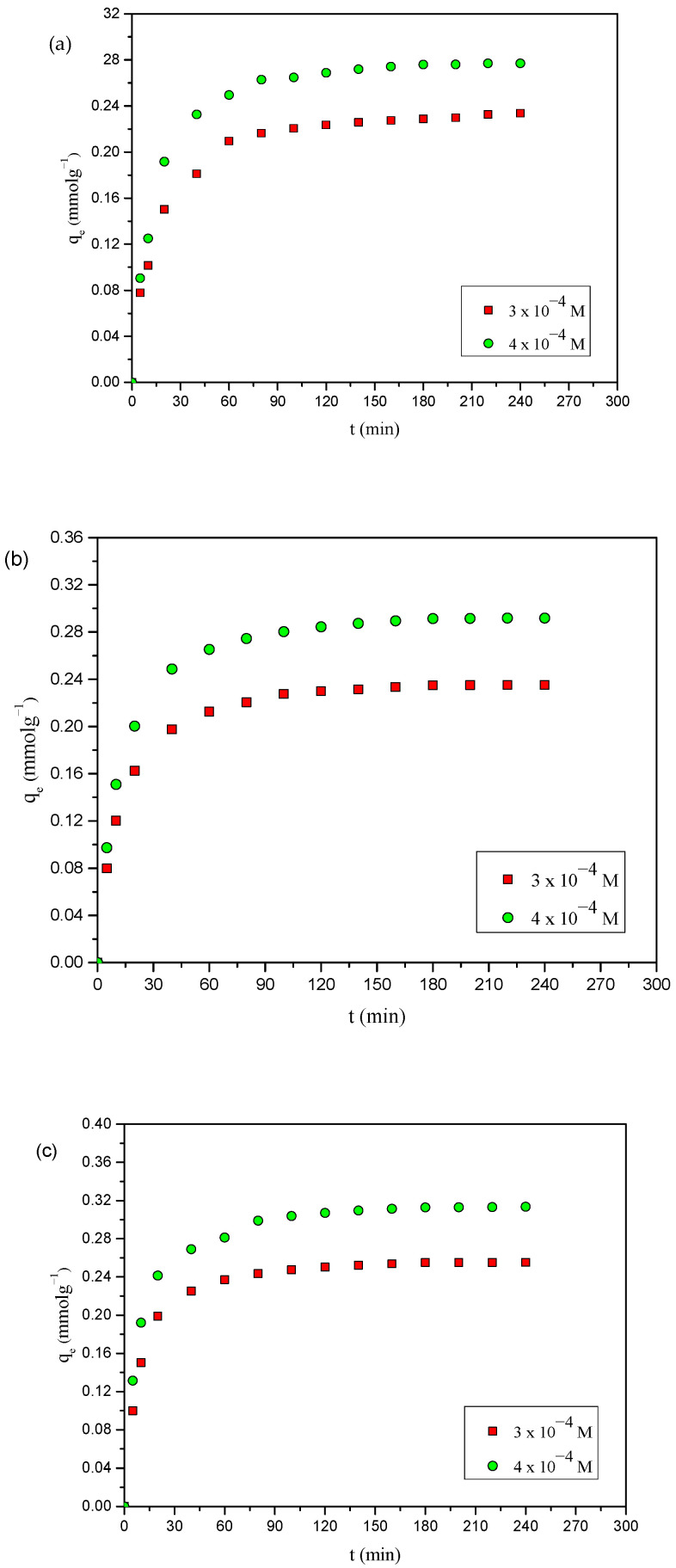
Effects of contact time and initial concentration on the adsorption of (**a**) phenol (**b**) 4-CP, and (**c**) 4-NP onto AC-HTPEFS at 25 °C.

Figure 1a–c show the adsorption of 4-NP, 4-CP, and phenol onto AC-HTPEFS as a function of contact time at different initial concentrations (3 × 10^−4^ and 4 × 10^−4^ M). The figure shows that, for all the phenols (4-NP, 4-CP, and phenol), changes in contact time have almost the same effect.

In the initial phase, the saturation curve shows a rapid increase, which slows as it progresses, eventually reaching a point of equilibrium [15]. The initial high uptake of phenols at the outset can be explained by the many available sites on the adsorbent surface (AC-HTPEFS), including free functional groups, active centers, and open pores. Filling the remaining vacant sites becomes progressively more difficult due to repulsion from the phenol molecules already adsorbed on the AC surface [16,17]. Figure 1a–c indicate that the adsorption equilibrium for 4-NP, 4-CP, and phenol was reached in 180 min. For further studies, the contact time was extended to 240 min.

The adsorption of 4-NP, 4-CP, and phenol was investigated at two different initial concentrations (3 × 10^−4^ and 4 × 10^−4^ mol dm^−3^), with the results presented in Figure 1a–c. Significantly, increasing the initial concentration leads to a corresponding increase in the adsorption capacity of phenols [18]. The increases in the initial concentrations delivered an enhanced driving force that retarded the mass transfer resistance of the phenols between the bulk and solid phases [19,20,21]. The influence of these parameters was not examined for the raw PEFS and HTPEFS due to the very low adsorption capacity presented by the phenols (4-NP, 4-CP, and phenol).

### 3.3. Adsorption Isotherms

**Figure 2:**
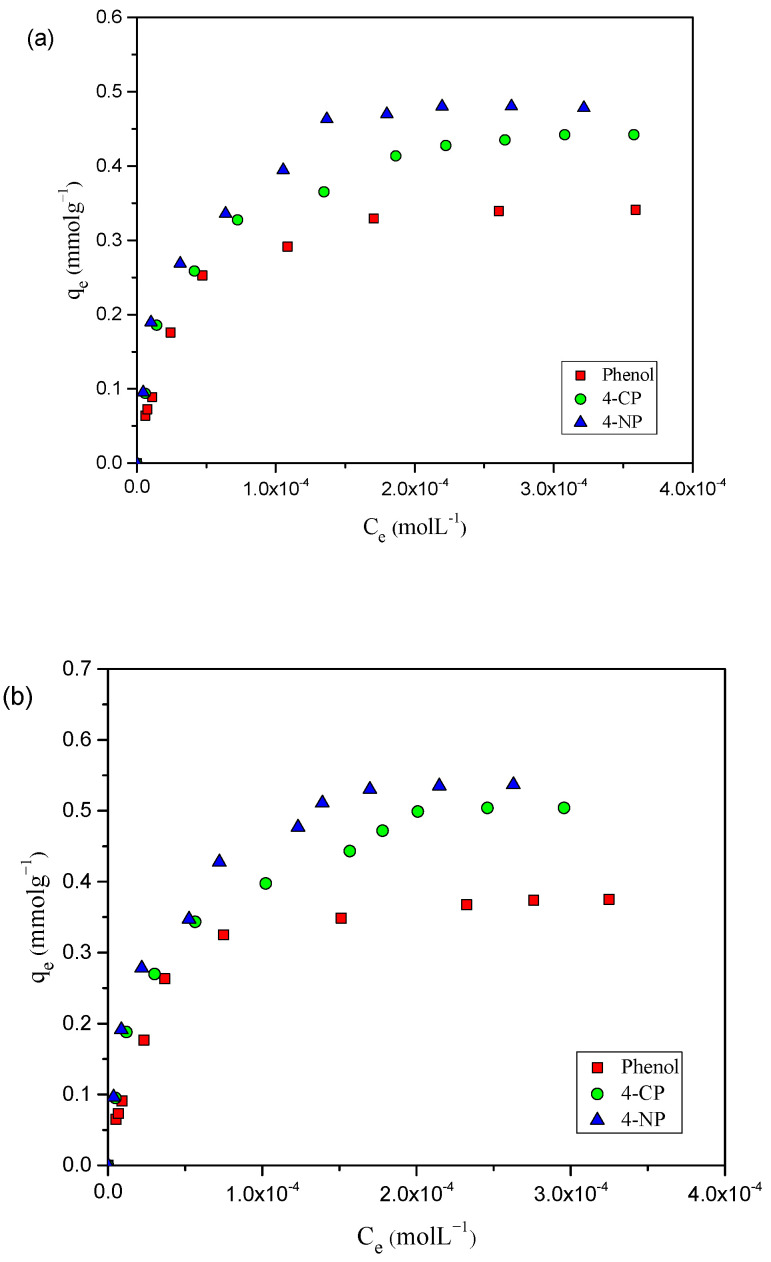

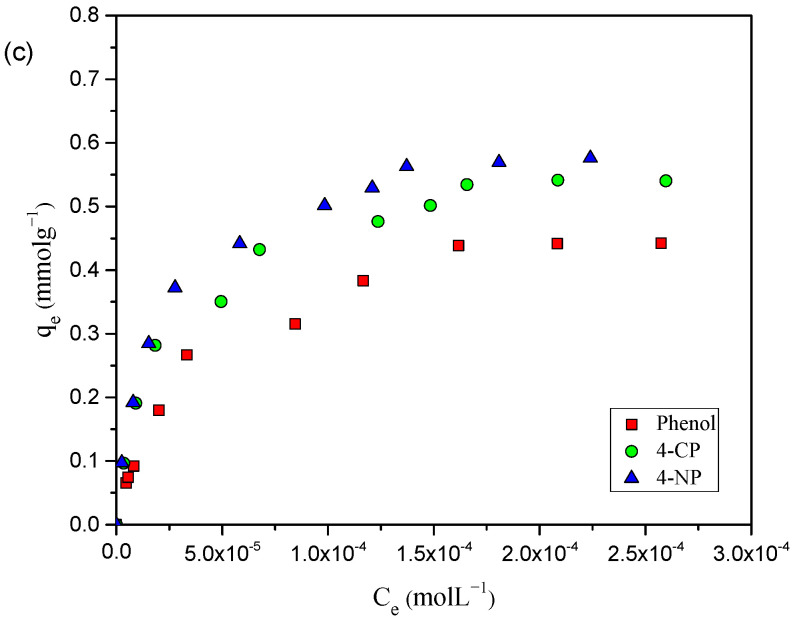
Adsorption isotherms of 4-NP, 4-CP, and phenol onto AC-HTPEFS at (**a**) 25 (**b**) 35, and (**c**) 45 °C.

The attainment of adsorption isotherms is very useful to describe the interaction between adsorbate and adsorbent. Adsorption isotherm studies provide important data concerning the properties, mechanism, and capacity of an adsorbent regarding adsorbate molecules [22]. The adsorption capacity of AC-HTPEFS for phenols at different temperatures is illustrated in Figure 2a–c. The experimental adsorption capacity at 25 °C on AC-HTPEFS followed the order of 4-NP ˃ 4-CP ˃ phenol, being 0.478, 0.442, and 0.341 mmol g^−1^, respectively.

**Figure 3:**
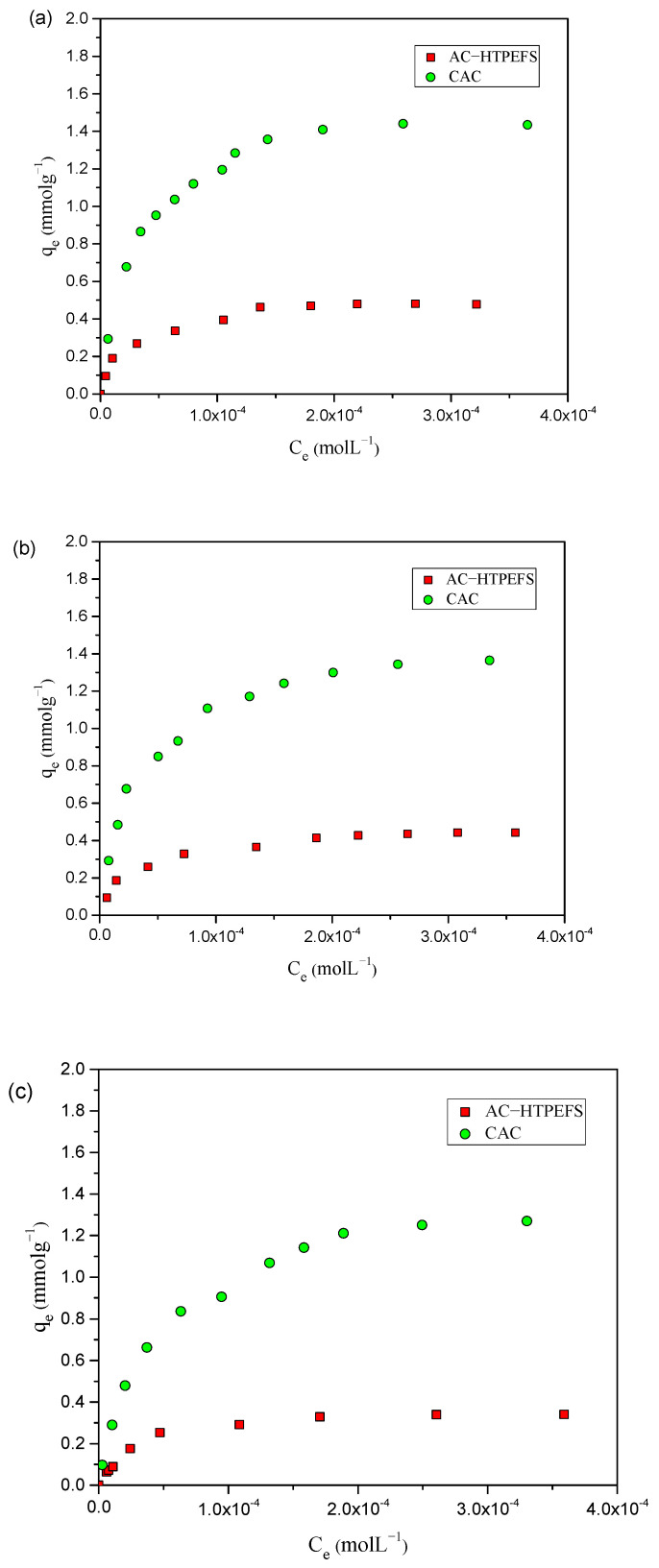
Adsorption isotherms of (**a**) 4-NP (**b**) 4-CP, and (**c**) phenol onto AC-HTPEFS and CAC at 25 °C.

The influence of temperature on adsorption experiments was evaluated between 25 and 45 °C, as illustrated in Figure 2a–c. The adsorption isotherms showed similar behavior regarding the quantity of phenols adsorbed, rising as the temperature increased. The amounts of phenols adsorbed were 0.478, 0.537, and 0.574 mmol g^−1^ for 4-NP, 0.442, 0.504, and 0.540 mmol g^−1^ for 4-CP, and 0.341, 0.375, and 0.442 for phenol at 25, 35, and 45 °C, respectively. Figure 2a–c indicate that 4-NP, 4-CP, and phenol adsorption was mainly an endothermic process. A comparison was conducted between the removal efficiencies of AC-HTPEFS and CAC at 25 °C, with results shown in Figure 3a–c.

Remarkably, a similar type of adsorption order for phenols was found with the CAC as well, with experimental adsorption capacities of 1.44, 1.35, and 1.26 mmolg^−1^ for 4-NP, 4-CP, and phenol, respectively. However, the higher values compared to AC-HTPEFS are clearly due to its greater surface area.

**Figure 4:**
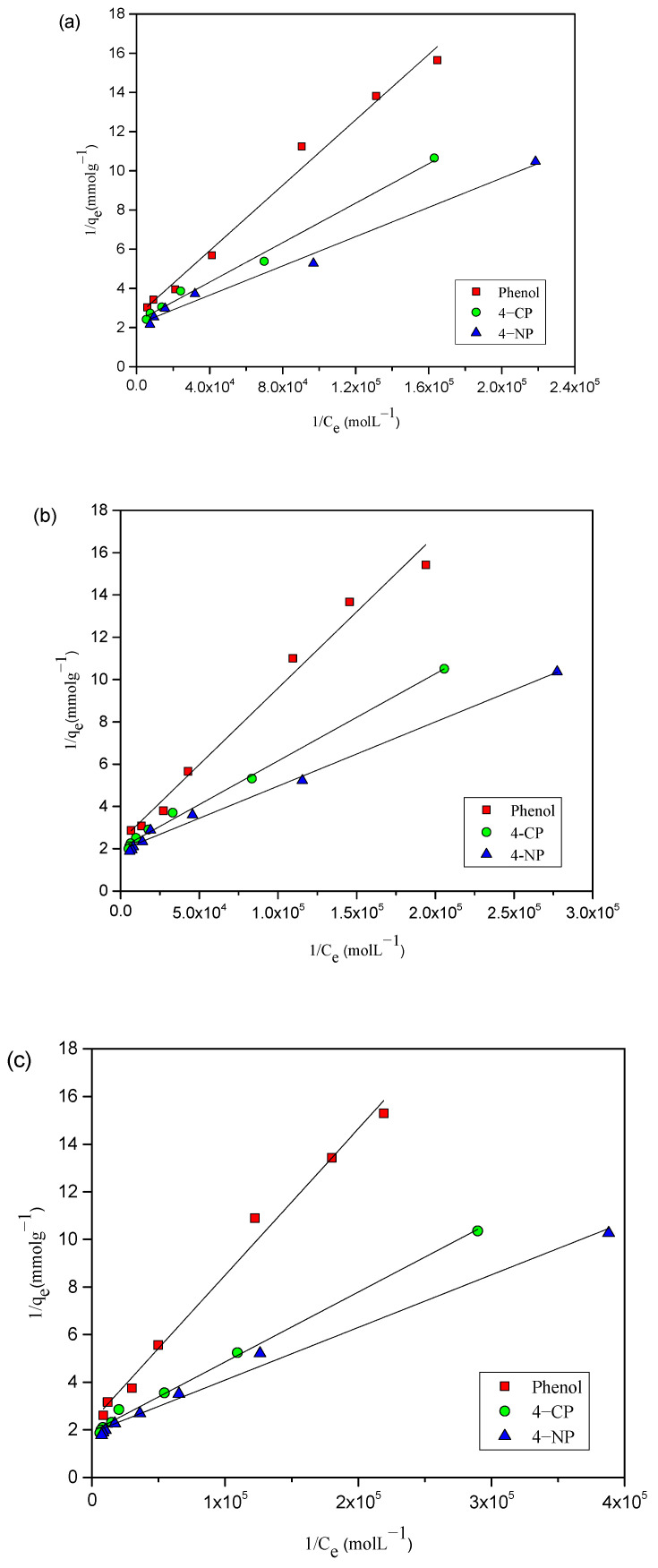
Langmuir adsorption isotherms of 4-NP, 4-CP, and phenol onto AC-HTPEFS at (**a**) 25 (**b**) 35, and (**c**) 45 °C.

The Langmuir, Freundlich, Temkin, and Dubinin–Radushkevich (D–R) isotherm models are commonly applied by researchers to model equilibrium adsorption data. The Langmuir [23] equation, the most frequently utilized model, is commonly expressed as

(2) $$\frac{1}{q_{e}}=\frac{1}{q_{m}}+\frac{1}{{q_{m}bC}_{e}}$$

where q_e_ (mmolg^−1^) represents amount of adsorbate (4-NP, 4-CP, and phenol) adsorbed at equilibrium concentration, b (L mol^−1^) represents Langmuir adsorption constant related to affinity of the adsorbate for the adsorbent, indicating a stronger affinity of the adsorbate for the adsorbent, q_m_ (mmol g^−1^) represents theoretical monolayer adsorption capacity, which provides insight regarding the maximum adsorption capacity toward the adsorbents, and C_e_ (mol L^−1^) represents equilibrium concentration of phenols. Langmuir plots were generated by plotting 1/q_e_ against 1/C_e_ for phenols (4-NP, 4-CP, and phenol) adsorbed at 25, 35, and 45 °C, as shown in Figure 4a–c. Further, the constant q_max_ and the Langmuir constant were calculated for 4-NP, 4-CP, and phenol and are provided in Table 1. The Langmuir constant (b) values provide insight into the adsorption intensity. Table 1 reveals that the values of b for 4-NP, 4-CP, and phenol are 5.78 × 10^4^, 4.57 × 10^4^, and 3.09 × 10^4^ Lmol^−1^, respectively, at 25 °C. This indicates that the AC-HTPEFS showed the maximum affinity for 4-NP and least affinity for phenol. Higher b values generally indicate stronger interactions between the phenols and AC-HTPEFS. For 4-NP, 4-CP, and phenol, the q_max_ values increase with temperature, reflecting the capacity of AC-HTPEFS to uptake more phenols as the temperature rises. For example, at 25 °C, the q_max_ for 4-NP is 0.463 mmol g^−1^, but, at 45 °C, it rises to 0.532 mmol g^−1^. This increase suggests that higher temperatures facilitate greater adsorption, indicating the suitability of the adsorbent for processes. The close match between q_max_ and q_exp_ values indicates the reliability of the Langmuir model. The correlation coefficients (R^2^) for 4-NP, 4-CP, and phenol in the Langmuir isotherm model are all close to 1, showing a high degree of fit between the q_max_ and q_exp_ values.

Furthermore, the shape of the isotherms enables predicting the favorability or unfavorability of an adsorption system. The characteristics of a Langmuir isotherm can be quantified using equilibrium parameter or dimensionless separation factor R_L_ [24], provided as follows:

(3) $$R_{L}=\frac{1}{1+{b\;C}_{0}}$$

where b (L mol^−1^) represents Langmuir constant and C_0_ (mol L^−1^) initial concentration. The value of R_L_ [24], which will be discussed later, provides insight into the nature of the adsorption process: R_L_ = 0—irreversible; 0 < R_L_ < 1—favorable; R_L_ > 1—unfavorable; R_L_ = 1—linear.

**Figure 5:**
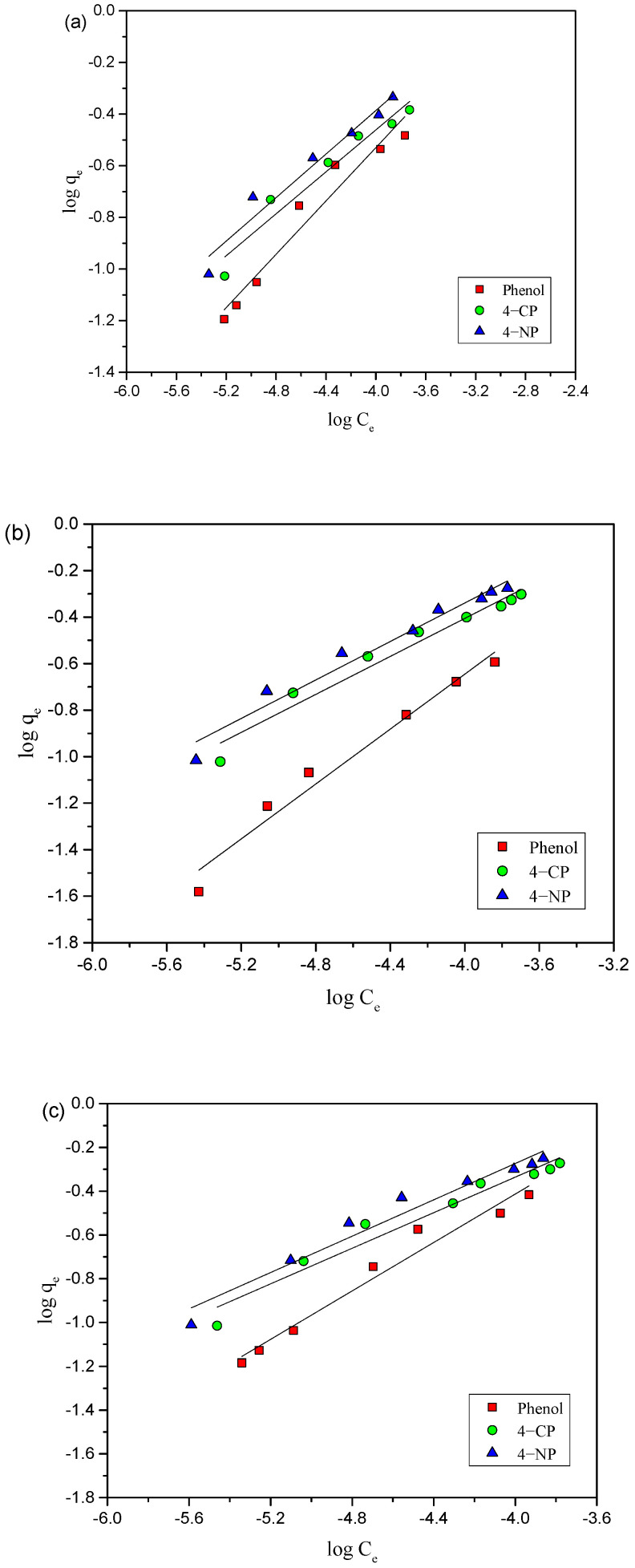
Freundlich adsorption isotherms of 4-NP, 4-CP, and phenol onto AC-HTPEFS at (**a**) 25 (**b**) 35, and (**c**) 45 °C.

Freundlich isotherm in general fits for heterogeneous surfaces [25] and is expressed as

log q_e_ = log K_F_ + (1/n) log C_e_ (4)

where K_F_ represents adsorption capacity and used to describe the adsorption characteristics of heterogeneous surfaces; q_e_ represents amount of the adsorbate (phenols) adsorbed at equilibrium concentration_;_ n represents correlation to the adsorption intensity and relates to the intensity of the adsorption or how favorable the adsorption process is. The plot created between log q_e_ and log C_e_ provides a straight line with K_F_ and n, which are obtained from intercept and slope, respectively, as shown in Figure 5a–c, at different temperatures (25, 35, and 45 °C), and results are included in Table 1.

The n values obtained from Freundlich model provide insights into the adsorption intensity and surface heterogeneity of the adsorbent. When n > 1, the adsorption process is considered to be favorable. Additionally, a higher n value suggests that the adsorbate molecules are more strongly adsorbed. Table 1 shows that the K_F_ values increased with rising temperature in each case (4-NP, 4-CP, and phenol), while the R^2^ values indicated a less favorable fit compared to Langmuir model.

The experimental data obtained were also analyzed by applying Temkin model [26] and can be provided as follows:

q_e_ = B_T_ lnA_T_ + B_T_ lnC_e_ (5)

where A_T_ is associated with the equilibrium binding constant, and it reflects the overall affinity of the adsorbent (AC-HTEFS) for the adsorbate (phenol). B_T_ = RT/ b_T_, where b_T_ is used to quantify the heat involved in the adsorption process; R represents ideal gas constant (8.314 J mol^−1^ K^−1^), and T indicates temperature (K) and A_T_ (L·mg^−1^) represents binding constant, indicating how strongly the adsorbate binds to the adsorbent. B_T_ (dimensionless) refers to heat of adsorption, providing insight into the energy changes occurring during the adsorption process.

**Figure 6:**
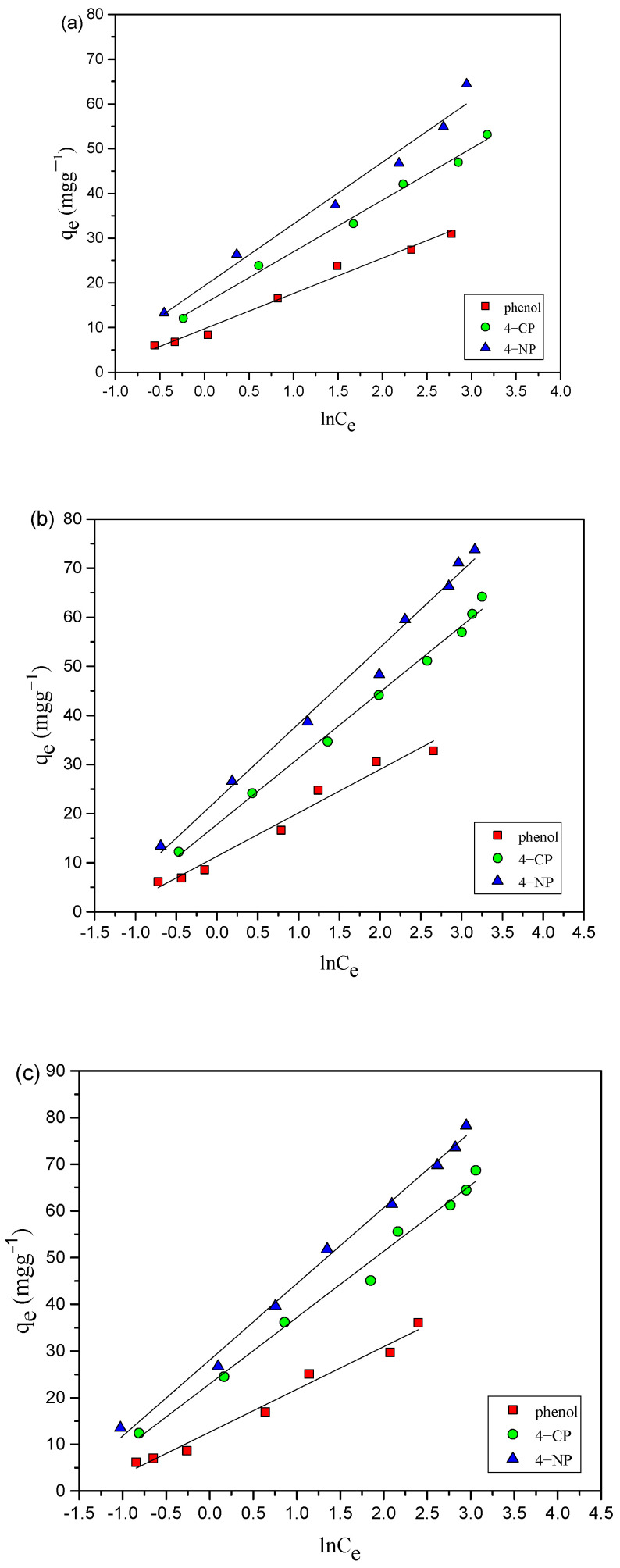
Temkin adsorption isotherms of 4-NP, 4-CP, and phenol onto AC-HTPEFS at (**a**) 25 (**b**) 35, and (**c**) 45 °C.

The graphs for Temkin model are shown in Figure 6a–c, and results in Table 1 indicate that the A_T_ (binding constant) values increase with increase temperature. This suggests that, at higher temperatures, the adsorbent (AC-HTPEFS) has a greater affinity for the adsorbate (phenol), which may be due to enhanced molecular interactions or increased surface activity of the adsorbent at elevated temperatures. Whereas, the B_T_ (heat of adsorption) values exhibit a decreasing trend as temperature increases, which shows that the adsorption process becomes less exothermic at higher temperatures, meaning less heat is released during adsorption. Moreover, the R^2^ values, which reflect the goodness of fit for the Temkin model, are comparatively lower than those for other isotherm models. This suggests that, while the Temkin model can describe the adsorption process, it is less accurate in predicting the adsorption behavior of phenols on AC-HTPEFS compared to other models.

To further evaluate the adsorption data for phenols on AC-HTPEFS, the D–R model [27] was applied. The model is outlined below as follows:

Ln q_e_ = ln q_m_ − βε^2^ (6)

**Figure 7:**
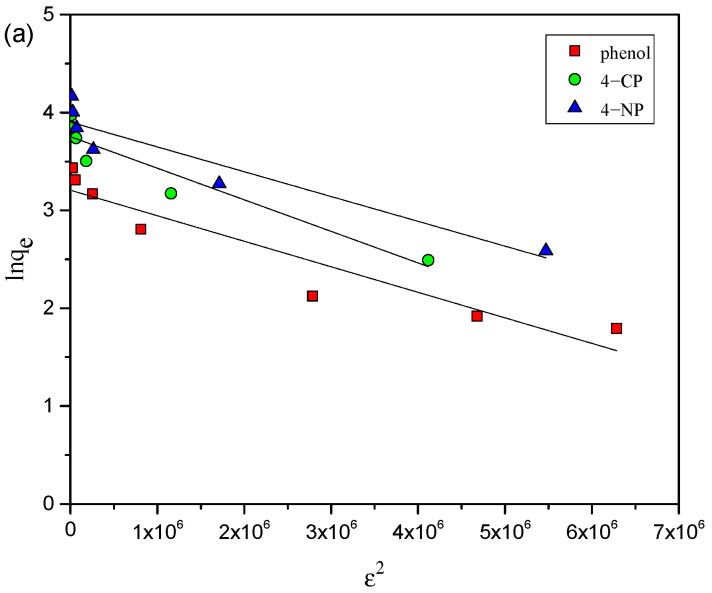

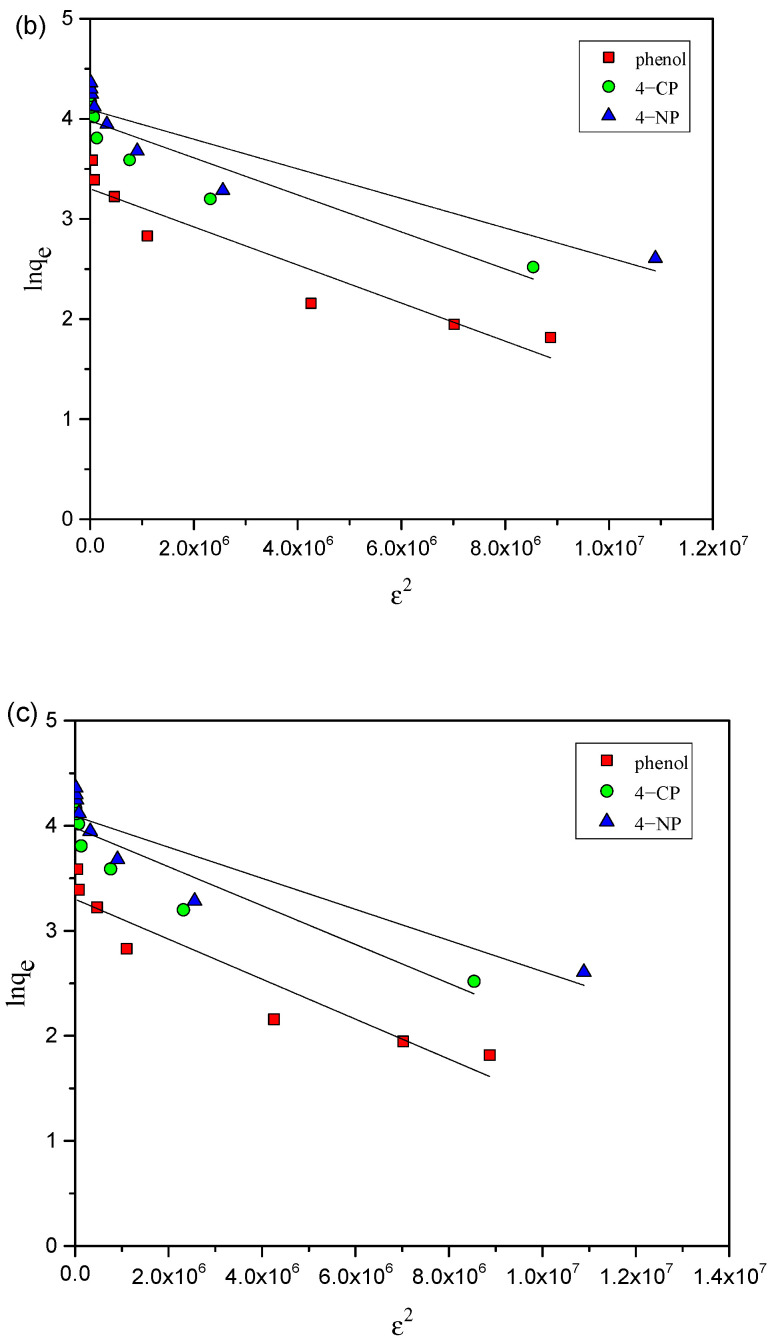
D–R adsorption isotherms of 4-NP, 4-CP, and phenol obtained on AC-HTPEFS at (**a**) 25 (**b**) 35, and (**c**) 45 °C.

ε = RT ln (1+ 1/C_e_) (7)

where ɛ represents Polanyi potential, accounting for the energetic characteristics of adsorption; q_m_ (mg g^−1^) indicates the D–R adsorption capacity; β_DR_ (mol^2^ k J^−2^) represents constant determined from mean free energy E (kJ mol^−1^) using the formula E = 1/√(2β_DR_). The D–R model parameters (Table 1), such as β_DR_ and q_m_, were derived from intercept and slope of the plots (Figure 7a–c) between lnq_e_ and ɛ^2^. For 4-NP, the q_m_ values for 4-NP, 4-CP, and phenol are 49.5, 42.7, and 24.7 mg g^−1^, respectively, at 25 °C, which are lower than the q_exp_ values. This shows that, while the AC-HTPEFS has a good capacity for phenols, the D–R model may not fully capture the maximum adsorption potential of adsorbent.

Moreover, the E values for 4-NP, 4-CP, and phenol range from 1.24 to 1.84 kJ mol^−1^. According to the D–R model, if E is less than 8 kJ mol^−1^, the adsorption process is dominated by physical adsorption (physisorption), involving weak van der Waals forces rather than chemical bonding.

Since the E values remain well below 8 kJ mol^−1^, it is clear that physical adsorption is the dominant mechanism. However, the small increase in E with temperature (for example, from 1.40 kJ mol^−1^ to 1.84 kJ mol^−1^ for 4-NP) may indicate a slightly stronger interaction at higher temperatures, but the adsorption process remains predominantly physical. Moreover, the lower R^2^ values, particularly for phenol, suggest that the D–R model might not fully account for the adsorption behavior, especially for systems involving more complex adsorbate interactions.

The correlation coefficient for the regression, R^2^, and the similarity between q_exp_ and q_max_ in Table 1 show that Langmuir model fits better to the experimental results than other models. Furthermore, the R_L_ values for all phenols, which range from 0 to 1, support the conclusion of favorable adsorption of phenols on AC-HTPEFS. In addition, there is a strong agreement between the theoretical monolayer adsorption capacity, as calculated from the Langmuir plot, and the experimental adsorption capacity. Therefore, results (better correlation coefficients) presented in Table 1 show that the Langmuir model offers the most precise and effective fit among the models evaluated.

The literature review shows that there are few studies on activated carbon prepared via hydrothermal treatment followed by activation for the removal of phenols. Therefore, Table 2 illustrates a comparative study of the monolayer adsorption capacities q_max_ of AC prepared by different methods derived from various precursors/raw materials for the removal of phenols (4-NP, 4-CP, and phenol). The values obtained in this study are in line with, and in some cases exceed, those reported for other samples in the literature, indicating that the AC-HTEFS produced here exhibits superior efficacy. These findings recommend that the AC-HTEFS prepared in this study has significant potential for commercial applications in phenol removal.

### 3.4. Mechanism of Adsorption of Phenols

Results obtained from adsorption isotherm clearly show that the q_max_ of 4-NP is higher when compared to 4-CP and phenol on the AC-HTPEFS. The adsorption process may be affected by factors including the adsorbate molecular size, solubility, and presence of the different substituting groups on phenols.

The molecular sizes of phenol, 4-CP, and 4-NP are 5.76 × 4.17 Å, 6.47 × 4.17 Å, and 6.84 × 4.17 Å, respectively [4]. Nevertheless, the pore diameter of adsorbent plays a key/remarkable role in adsorption. The literature shows that adsorbents have optimal adsorption properties when their pore width is 1.7 to 3 times greater than the size of the adsorbate molecules [34]. As per theoretical dimensions, the adsorption of phenol should be higher in comparison to 4-NP and 4-CP. The experimental results did not follow this tendency, leading to the belief that the phenolic compounds’ adsorption is not a simple physical process but involves other specific interactions [35].

**Figure 8:**
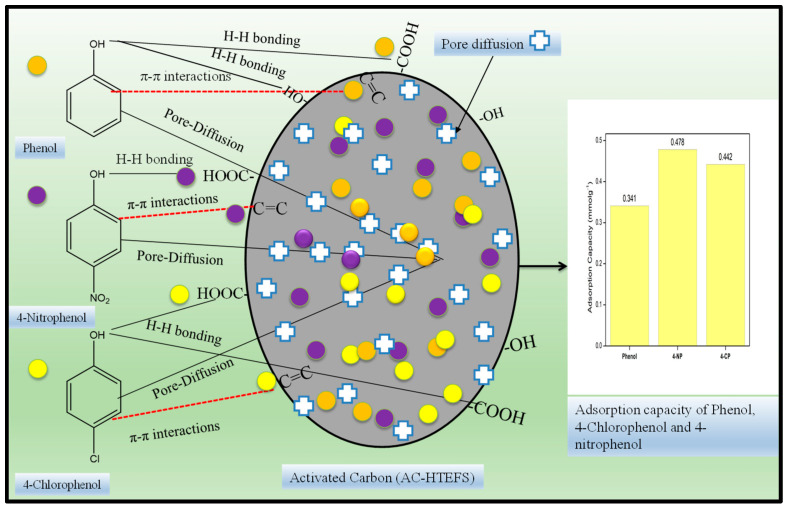
The adsorption mechanism for the removal of phenols regarding AC-HTPEFS.

In the adsorption of organic molecules that have a benzene ring or C=C in their structure, π−π interaction played an important role (Figure 8). The π electron of the organic molecule will interact with the π electron of the benzene ring of the AC [36]. In this study, the selected phenols have a benzene ring in their structure; hence, it could be speculated that, in the adsorption of these phenols on the AC, π−π interaction will play an important role. Furthermore, the introduction of the different substituent groups to the aromatic ring will affect strength of these π−π interactions by affecting the electron density of the aromatic system. The electron-withdrawing groups reduce the density of the electron and thus will increase the interaction since it will reduce electrostatic repulsion [37]. Chloro and nitro are electron-withdrawing groups, and the nitro group is stronger than chloro; hence, the π−π interactions will be stronger between adsorbent (AC-HTPEFS) and 4-NP when compared with 4-CP [4]. On the other hand, phenol has fewer interactions as in its structure no electron-withdrawing groups are present. This would explain why the maximum adsorption capacity is exhibited by 4-NP, followed by 4-CP and phenol.

It is also worth mentioning that the different oxygen-containing functional groups found on the surface of AC-HTPEFS as discussed elsewhere [14] will also form hydrogen bonds with the hydroxyl group of the phenols. Phenol (4-NP, 4-CP, and phenol) adsorption is also affected by hydrophobic interactions, with more hydrophobic adsorbates showing a greater tendency to be adsorbed and remained on the surface or within the pores of AC-HTPEFS [4]. For the adsorption of 4-NP, 4-CP, and phenol onto AC-HTPEFS, the hydrophobicity of the solute is influenced by its solubility, and greater solubility generally results in lower hydrophobicity. It is further added that the solubility values in water at 25 °C of 4-NP, 4-CP, and phenol are provided as 1.69, 27, and 93 g L^−1^, respectively. Thus, the hydrophobicity will be highest for 4-NP and lowest for phenol. Based on all reported properties of the phenolic compounds evaluated, the maximum adsorption capacity must follow the order 4-NP ˃ 4-CP ˃ phenol.

### 3.5. Thermodynamics of Phenol Adsorption

The thermodynamic aspects of adsorption process, viz. free energy change (ΔG^o^), enthalpy change (ΔH^o^), and entropy change (ΔS^o^), were assessed using the following equations:

ΔG^o^ = − R T ln (b) (8)

(9) $$\ln\;b=-\frac{∆H{}^{\circ}}{RT}+\frac{∆S{}^{\circ}}{R}$$

**Figure 9:**
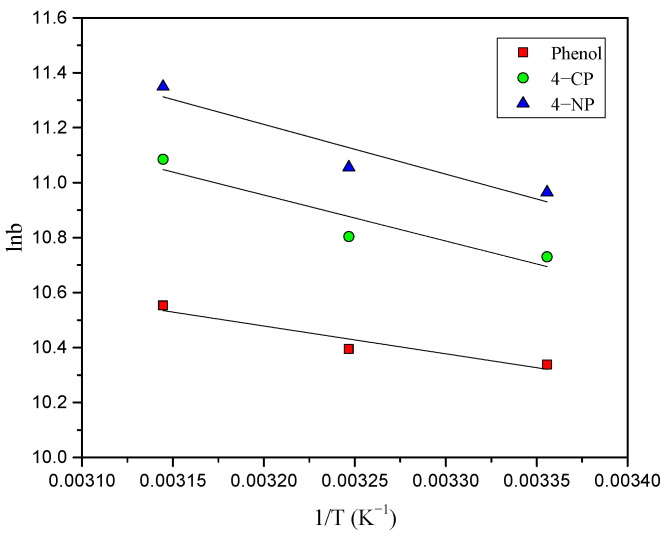
Van’t Hoff plots for the adsorption of 4-NP, 4-CP, and phenol onto AC-HTPEFS.

The values of ΔS^o^ and ΔH^o^ were determined from intercept and slope of the plot (Figure 9) between ln b vs. 1/T using the Van’t Hoff equation (Equation (9)), and results are provided in Table 3.

The ΔH^o^ values of 4-NP, 4-CP, and phenol are 15.1, 13.9, and 8.3 kJ mol^−1^, respectively, confirming that the adsorption is an endothermic process. The low and negative values of ΔG^o^ obtained show that the adsorption process is spontaneous and favorable. The positive ΔS^o^ values show the presence of more degrees of randomness of phenol molecules at solid/solution interface [4].

### 3.6. Kinetics Studies

**Figure 10:**
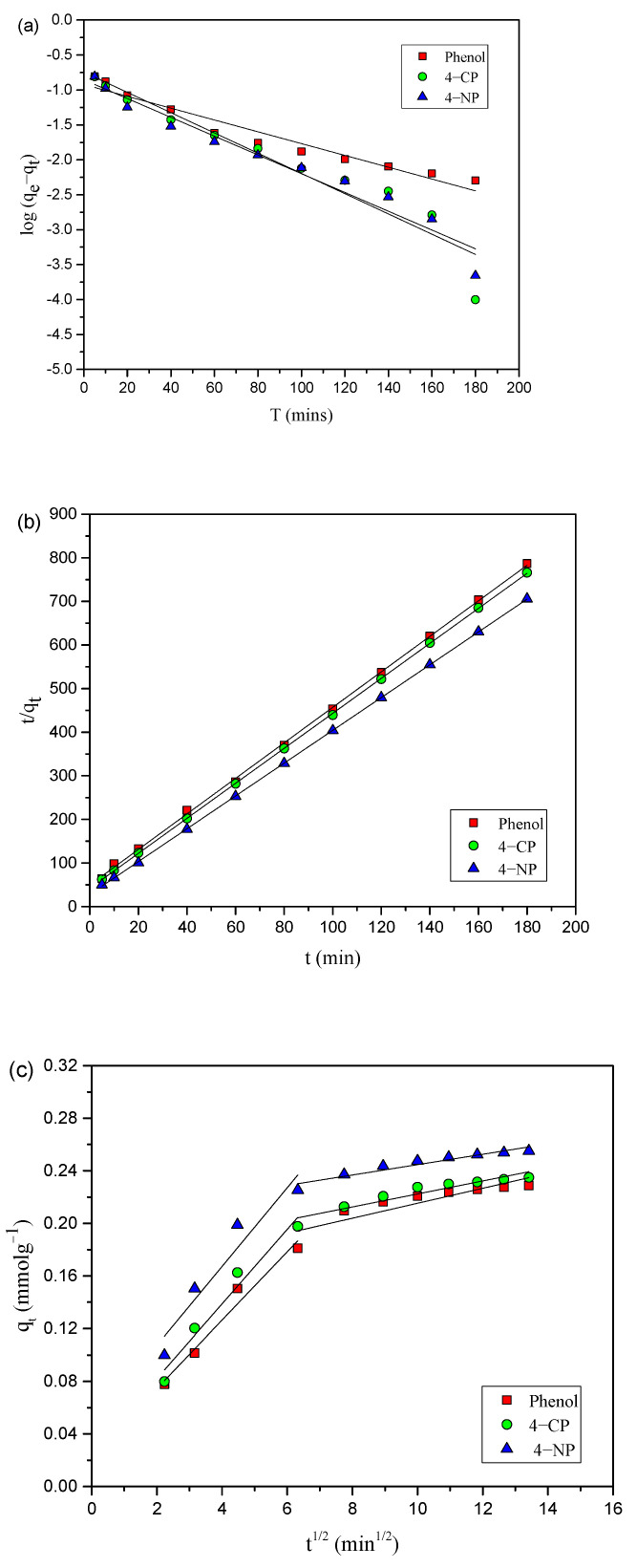
(**a**) PFO, (**b**) PSO, and (**c**) IPD kinetic plots for the adsorption of 4-NP, 4-CP, and phenol onto AC-HTPEFS at 3 × 10^−4^.

Kinetics studies were undertaken to explore the dominant adsorption mechanisms, including chemical reaction kinetics and mass transfer. Studies were conducted on the adsorption of 4-NP, 4-CP, and phenol onto AC-HTPEFS over a 240 min period. Well-known kinetics models, viz. PFO [38], PSO [39], and IPD [40], were applied. The PFO kinetics model provided by Lagergren is represented by Equation (10) and was used to analyze the experimental data.

(10) $$\log\;(q_{e}-q_{t})=\log\;q_{e}-\frac{k_{1}}{2.303}t$$

where k_1_ represents the Lagergren adsorption rate constant; q_t_ and q_e_ represent amounts adsorbed at any time t and at equilibrium, respectively. Plots (Figure 10a) of log (q_e_ − q_t_) vs. t for 4-NP, 4-CP, and phenol were made. The constants k_1_ and q_e(cal)_, presented in Table 4, were determined from slope and intercept, respectively. It was observed that q_e(exp)_ values deviate from the q_e(cal)_ values obtained from the linear plot for 4-NP, 4-CP, and phenol.

The PSO [39] model was also applied to the experimental data, and the equation is represented as

(11) $$\frac{t}{q_{t}}=\frac{1}{k_{2}q_{e}^{2}}+\frac{1}{q_{e}}\;t$$

where k_2_ represents the equilibrium rate constant; q_e_ and q_t_ represent amounts of phenols (4-NP, 4-CP, and phenol) adsorbed onto AC-HTPEFS at equilibrium and time (t), respectively. The graphs (Figure 10b) were plotted as t/q_t_ versus time, and straight lines were obtained for all phenols. The values of k_2_ and q_e_ obtained from Figure 10b are shown in Table 4. It is evident that the calculated q_e(cal)_ from the plot aligns closely with the experimental q_e(exp)_. In addition to the q_e(cal)_, the correlation coefficient (R^2^) values obtained for the applied kinetics models are shown in Table 4. The data in Table 4 indicate that the R^2^ values for the PFO model are lower than in the PSO model, suggesting that the adsorption of phenols on adsorbent (AC-HTPEFS) follows PSO kinetics model.

The IPD model, or Weber–Morris plot [40], was applied to explore the adsorption mechanism for the removal of phenols onto AC-HTPEFS, and the rate constant was calculated as

q_t =_ K_id_·t^1/2^ + C (12)

where C represents constant related to boundary layer thickness; k_id_ represents IPD rate constant. To assess the IPD model, q_t_ versus t^1/2^ plots (Figure 10c) were constructed. The plots in Figure 10c for phenols reveal that the adsorption process comprises two stages: the initial stage of film diffusion followed by IPD [41]. The rate constants Kp_1_ and Kp_2_ for the first and second stages of IPD, respectively, and the corresponding intercepts C_1_ and C_2_, were extracted from q_t_ versus t^1/2^ plots and are listed in Table 4. The observation that Kp_2_ values are lower than Kp_1,_ for all phenols suggests that IPD is a key rate-limiting step. Figure 10c shows that neither linear plot intersects the origin, which points to the involvement of additional rate-controlling factors beyond IPD [41]. Consequently, the adsorption of 4-NP, 4-CP, and phenol onto AC-HTPEFS involves a complex interplay of both surface diffusion and IPD in determining the rate-limiting step.

## 4. Conclusions

In this study, the AC-HTPEFS was prepared by heating the HTPEFS in an oxidizing atmosphere (air) at 400 °C for 90 min. The raw material, the AC-HTPEFS, and a CAC were tested on the removal of three phenols (4-NP, 4-CP, and phenol). It was found that a higher initial phenol concentration and high temperature increased the adsorption capacity. The phenol and substituted phenol (4-NP and 4-CP) adsorption isotherms obtained regarding the AC-HTPEFS were effectively described by the Langmuir model. The lower solubility in water of 4-NP compared to 4-CP and phenol confirm its higher hydrophobicity, which agrees with the following adsorption sequence: 4-NP > 4-CP > phenol, aligning with the π−π interaction order. The thermodynamic analysis further revealed that the adsorption of three phenols onto AC-HTPEFS is an endothermic and spontaneous process, where the physisorption is predominant. The kinetic data fitting results suggested that the removal of 4-NP, 4-CP, and phenol onto AC-HTPEFS followed the PSO kinetics model based on a complex process, where the IPD and surface diffusion contribute to the rate-limiting step.

## Figures and Tables

**Table 1:** Adsorption isotherm parameters for the adsorption of 4-NP, 4-CP, and phenol onto AC-HTPEFS.

Adsorbate	4-Nitrophenol			4-CP			Phenol		
Experiments and Models Applied	Temperature			Temperature			Temperature		
	25 °C	35 °C	45 °C	25 °C	35 °C	45 °C	25 °C	35 °C	45 °C
Experimental data q_exp_ (mmol g^−1^) mg g^−1^	0.478 66.5	0.537 74.7	0.574 80.1	0.442 56.9	0.504 64.8	0.540 69.5	0.341 32.1	0.375 35.3	0.442 41.6
Langmuir parameters									
q_max_ (mmol g^−1^)	0.463	0.520	0.532	0.434	0.493	0.522	0.387	0.423	0.425
b (L mol^−1^)	5.78 × 10^4^	6.33 × 10^4^	8.49 × 10^4^	4.57 × 10^4^	4.92 × 10^4^	6.52 × 10^4^	3.09 × 10^4^	3.27 × 10^4^	3.83 × 10^4^
R^2^	0.988	0.994	0.992	0.992	0.996	0.996	0.988	0.985	0.990
Freundlich parameters									
K_f_ (mmolg^−1^) (molL^−1^)^1/n^	20.0	20.6	24.5	12.7	16.9	19.6	34.3	56.9	63.5
n	2.37	2.42	2.41	2.46	2.45	2.46	1.94	1.81	1.80
R^2^	0.959	0.966	0.960	0.949	0.964	0.957	0.949	0.949	0.968
Temkin parameters									
A_T_ (L mg^−1^)	4.05	4.33	5.61	3.78	3.75	5.06	3.44	3.60	3.97
b_T_ (KJ mol^−1^)	0.179	0.159	0.152	0.214	0.184	0.174	0.314	0.280	0.271
R^2^	0.978	0.987	0.993	0.992	0.994	0.989	0.985	0.973	0.981
D–R parameters									
q_m_ (mg g^−1^)	49.5	57.8	59.8	42.7	50.2	53.5	24.7	26.3	27.1
E (KJ mol^−1^)	1.40	1.53	1.84	1.24	1.36	1.64	1.38	1.49	1.62
R^2^	0.896	0.861	0.834	0.905	0.860	0.859	9.901	0.893	0.899

**Table 2:** Comparison of monolayer adsorption capacity of activated carbons prepared by different methods from different raw materials.

Precursor	Methods of Preparation of Activated Carbon	Pollutant	Surface Area (m^2^g^−1^)	Adsorption Capacity q_max_ (mgg^−1^)	Reference
Rice husk	Hydrothermal carbonization at 300 °C; physical activation at 800 °C in CO_2_	Phenol	358	39.30	[28]
Coffee grounds	Carbonization and chemical activation with ZnCl_2_ + H_3_PO_4_ at 600 °C	Phenol	640	3.22	[29]
Avocado kernels	Carbonization, activation at 1173 K in CO_2_	Phenol	206	90	[30]
*Phyllanthus emblica* fruit stone	Hydrothermal carbonization at 121 °C; physical activation in air at 400 °C	Phenol	569	32.1	This study
Orange Peel	Carbonization and chemical activation with orthophosphoric acid at 350 °C	4-nitrophenol	540.61	73.35	[31]
Carrot dross	Activation at 500 °C in air atmosphere	4-nitrophenol	447	91	[32]
*Phyllanthus emblica* fruit stone	Hydrothermal carbonization at 121 °C; physical activation in air at 400 °C	4-nitrophenol	569	66.5	This study
Pea nut husk	Carbonization and chemical activation with (NH_4_)_2_HPO_4_ at 450 °C	4-chlorophenol	499.9	98.2	[33]
*Phyllanthus emblica* fruit stone	Hydrothermal carbonization at 121 °C; physical activation in air at 400 °C	4-chlorophenol	569	56.9	This study

**Table 3:** Thermodynamic parameters for the adsorption of 4-NP, 4-CP, and phenol onto AC-HTPEFS.

Phenols	Temperature (°C)	ΔG^o^ (kJ mol^−1^)	ΔS^o^ (J mol^−1^ K^−1^)	ΔH^o^ (kJ mol^−1^)
4-NP	25 35 45	−27.2 −28.3 −30.0	141	15.1
4-CP	25 35 45	−26.6 −27.7 −29.3	136	13.9
Phenol	25 35 45	−25.6 −26.6 −27.9	114	8.3

**Table 4:** Kinetic parameters for the adsorption of 4-NP, 4-CP, and phenol onto AC-HTPEFS.

Phenols	C_o_ (mol L^−1^)	q_e(exp)_ (mmol g^−1^)	PFO			PSO			IPD					
			q_e (cal)_ (mmol g^−1^)	K_1_ (min^−1^)	R^2^	q_e(cal)_ (mmol g^−1^)	K_2_ (g·mmol^−1^·min^−1^)	R^2^	K_p1_	C_1_	R^2^	K_p2_	C_2_	R^2^
4-NP	3 × 10^−4^	0.260	0.140	3.09 × 10^−2^	0.958	0.266	0.505	0.999	3.00 × 10^−2^	4.73 × 10^−2^	0.922	3.90 × 10^−3^	0.2052	0.919
4-CP	3 × 10^−4^	0.240	0.180	3.34 × 10^−2^	0.922	0.249	0.385	0.999	2.37 × 10^−2^	4.13 × 10^−2^	0.946	3.80 × 10^−3^	0.1866	0.921
Phenol	3 × 10^−4^	0.234	0.119	1.96 × 10^−2^	0.949	0.245	0.334	0.999	2.39 × 10^−2^	2.96 × 10^−2^	0.978	3.30 × 10^−3^	0.1862	0.955

## Data Availability

The data of this study are available upon reasonable request to the corresponding authors.
